# Supplementary figures and images for: Anemia biomarkers and mortality in hemodialysis patients with or without diabetes: A 10-year follow-up study
Source: PLoS One. 2023 Jan 31;18(1):e0280871. doi: 10.1371/journal.pone.0280871 (PMC9888689; doi:10.1371/journal.pone.0280871)

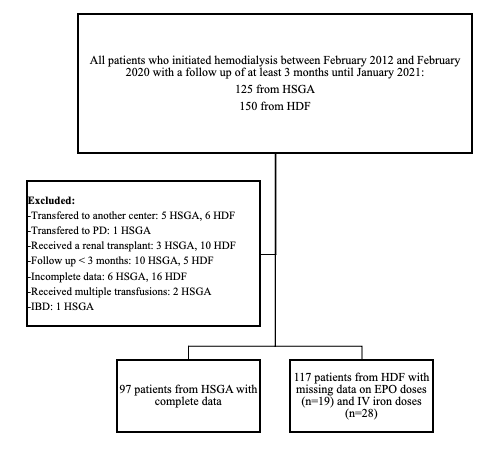

Supplement: S1 Fig — (TIFF) [file pone.0280871.s001.tiff]

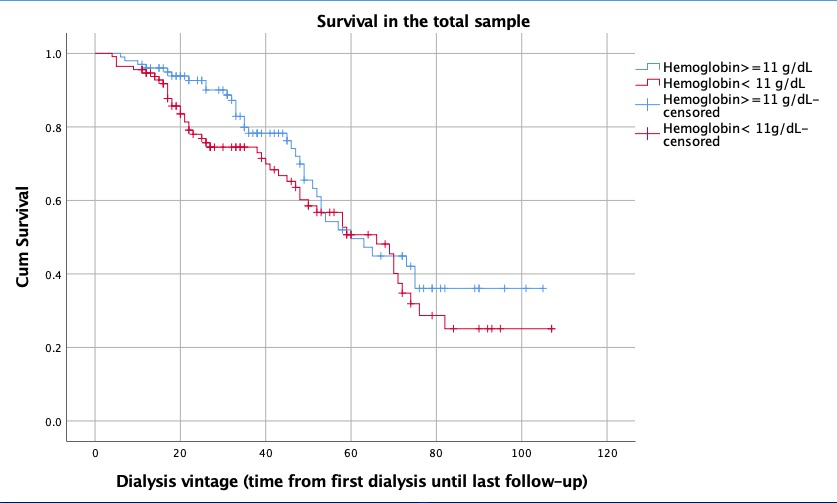

Supplement: S2 Fig — (TIFF) [file pone.0280871.s002.tiff]
