# Supplementary material for: Anemia biomarkers and mortality in hemodialysis patients with or without diabetes: A 10-year follow-up study
Source: PLoS One. 2023 Jan 31;18(1):e0280871. doi: 10.1371/journal.pone.0280871 (PMC9888689; doi:10.1371/journal.pone.0280871)
Supplement: S1 File — (PDF) [file pone.0280871.s003.pdf]

HOTEL-DIEU DE FRANCE
Centre Hospitalier de l'Université Saint-Joseph de Beyrouth

Université Saint-Joseph de Beyrouth 1875
جامعة القدّيس يوسف في بيروت

Comité d'éthique

Beirut, March 11, 2021

Doctor Mabel AOUN
Department of Nephrology
Hôpital Saint-Georges Ajaltoun

Tfem/2022/9

Dear colleague,

During its meeting on March 9, the Ethics Committee examined the thesis protocol for medical studies MD student Miss Jihane ASMAR, entitled "Aossication between the risk of mortality and the figures for hemoglobin and iron markers in hemodialysis patients", and done under your supervision.

After deliberation, the Committee unanimously considers that this project raises no ethical objection; it therefore gives you its approval for the conduct of this research.

In addition, I inform you that since the study is retrospective it will be unnecessary to collect the patients' consent form. Please make sure you send us the agreements of the Saint Georges Hospital Ajaltoun and the Saint Joseph Hospital Dora .

The Committee was composed of Professors Marie-Helene GANNAGE, Sami RICHA and Michel SCHEUER. The Ethical Committee of Hotel Dieu de France acts in agreement with good clinical practice (GCP) and following a written operating protocol.

Best regards,

P.O. Professor Michel SCHEUER

The Committee secretary

Nancy Ab[illegible]

HOTEL-DIEU DE FRANCE HDF Comité d'éthique

www.hdf.usj.edu.lb

Hôtel-Dieu de France
B.P. 16-6830 Achrafieh, Beyrouth - Liban
tél. +961-1-421 229
cue@usj.edu.lb
