# Supplementary material for: Anemia biomarkers and mortality in hemodialysis patients with or without diabetes: A 10-year follow-up study
Source: PLoS One. 2023 Jan 31;18(1):e0280871. doi: 10.1371/journal.pone.0280871 (PMC9888689; doi:10.1371/journal.pone.0280871)
Supplement: S2 File — (PDF) [file pone.0280871.s004.pdf]

HDF
HOTEL-DIEU DE FRANCE
Centre Hospitalier de l'Université Saint-Joseph de Beyrouth

USJ 1875
Université Saint-Joseph de Beyrouth
جامعة القديس يوسف في بيروت

Comité d'éthique

Pr Sami Richa, *Président*
Pr Michel Scheuer s.j.
Pr Georges Abi Tayeh
Pr Claudia Khayat
Pr Nasri Diab
Mlle Soha Abdel Malak
Dr Jad Habib
Mme Hyam Kahi
Pr Ronald Moussa
Pr Marek Cieslik s.j.
Dr May Fakhoury
M. Ayad Wakim

Beyrouth, le 11 mars 2021

Madame le Docteur Mabel AOUN
Service de nephrology
Hôpital Saint-Georges Ajaltoun

Tfem/2022/9

Chère Collègue,

Lors de sa réunion du 9 mars, le Comité d'éthique a examiné le protocole d'étude de la thèse de fins d'études médicales de Mlle Jihane ASMAR, intitulée « Aossication entre le risque de mortalité et les chiffres d'hémoglobine et des marqueurs du fer chez les hémodialysés » réalisée sous votre direction.

Après en avoir délibéré, le Comité restreint estime à l'unanimité que cette étude ne soulève aucune objection d'ordre éthique, il vous notifie donc bien volontiers son accord. Par ailleurs, je vous informe de la non nécessité de faire signer un formulaire d'information et de consentement dans le cas d'une étude rétrospective et à nous faire parvenir l'accord des l'hôpitaux S-Georges Ajaltoun et St-Joseph Dora.

Le Comité restreint était composé des Professeurs Marie-Hélène GANNAGÉ, Sami RICHA et Michel SCHEUER. Le Comité agit en concordance avec les « Bonnes Pratiques Cliniques » (GCP) décrites dans la « Déclaration d'Helsinki » (version d'octobre 2013) et les « Lignes directrices internationales d'éthique pour la recherche biomédicale impliquant des sujets humains » du Conseil des Organisations internationales des Sciences médicales (CIOMS) avec la collaboration de l'Organisation mondiale de la santé (OMS).

Avec mes meilleures salutations.

HOTEL-DIEU DE FRANCE * HDF * comité d'éthique *

Pr. Michel SCHEUER
Secrétaire du Comité

cc. Marie-Hélène GANNAGÉ

N.B. Merci d'aviser le CÉ de toute modification au protocole de recherche pouvant avoir une incidence sur le plan éthique et de l'informer des conclusions générales et des résultats de l'étude.

Hôtel-Dieu de France
B.P. 16-6830 achrafieh, beyrouth - liban
tél. +961-1-421 229
cue@usj.edu.lb
