# Supplementary material for: Anemia biomarkers and mortality in hemodialysis patients with or without diabetes: A 10-year follow-up study
Source: PLoS One. 2023 Jan 31;18(1):e0280871. doi: 10.1371/journal.pone.0280871 (PMC9888689; doi:10.1371/journal.pone.0280871)
Supplement: Attachment — Submitted filename: Response to reviewers_Anemia .docx [file pone.0280871.s006.docx]

Dear Editor,

Thank you for giving us the opportunity to submit a revised version of our manuscript.

Please find below a point-by-point response to reviewers.

**Reviewer #1:**

Jihane Asmar et al. investigated the impact of Hb, ferritin, and TSAT on mortality of HD patients with or without diabetes. Log rank test showed an association between Hb ≥ 11 g/dL and better survival in patients without diabetes but not in patients with diabetes. Hb, ferritin and age were independent factors associated with mortality in patients without diabetes. Although the enrolled patients were relatively small numbers, however, the study has strength such as long-term follow-up and usage of the average data of all parameters of Hb during entire observation period.

Major comments
1. Reviewer’s comment: In page 6 lines 1-2, did the authors investigate the compliance rate for the KDIGO guideline for anemia management in the current investigation.

Authors’ reply: Thank you for your comment. It would have been interesting to assess the compliance of nephrologists to KDIGO guidelines. However, evaluating the compliance rate to KDIGO was not the aim of this study. This needs to be conducted as a study on its own. But we know that all nephrologists in the two units follow these guidelines. We amended the statement in the methods as follows: “the six nephrologists of these two units have been following the KDIGO guidelines for anemia management”. In addition, we amended the description of Hb categories in Table 2 where we showed that ~10% of patients had a Hb<10 and ~8% had a Hb≥12 and this is very consistent with the KDIGO recommendations.

2. Reviewer’s comment: In page 6 line 3, are 3 months treatment of HD enough for the evaluation of the survival of the patients?

Authors’ reply: Thank you for your comment. Survival analysis assesses the expected duration of time until death occurs. In the general population, three months might seem like a short period of time but in hemodialysis patients with very reduced survival, including patients starting three months of follow-up is quite acceptable and most studies of risk factors of mortality in hemodialysis start at 3 months. Anyways, the dialysis vintage of our group of patients varied between a minimum of 4 months and a maximum of 107 months with a mean of 40 months; only 5 patients had a follow-up less than 6 months. We added the latter to the manuscript.

3. Reviewer’s comment: In page 7 lines 1-2, the authors should describe the percentage of missing data and how did the authors perform the statistical analyses for missing data.

Authors’ reply: Thank you for your comment. We did not have missing data for the outcome variables or the hemoglobin variable or other laboratory values. Therefore, all the analysis done was based on full data, except for the regression model 3 in Table 5 that included the two variables where there was 8.1% and 13.1% of missing data for doses of EPO and IV iron respectively. The Little’s MCAR test revealed a p-value of 0.003. Therefore, we did a multiple imputation and amended the results of this model. We updated as well the statistical analysis paragraph accordingly.

4. Reviewer’s comment: In page 8 line 18, are there any differences in survival between twice-weekly and triple-weekly HD patients?

Authors’ reply: Thank you for your comment. This study did not aim to compare survival between twice and thrice-weekly hemodialysis. But based on the reviewer’s suggestion, we ran a log rank test to compare these two groups and it revealed a non-significant *p* value of 0.65.

5. Reviewer’s comment: In page 9, Table 1, the rate of cancer in cause of death is very low. Is there any explanation for it? Were the enrolled HD patients relatively younger?

Authors’ reply: The death due to cancer is 6.3% which is aligned with a lot of data worldwide. The major causes of death in hemodialysis are cardiovascular, sudden cardiac arrest and infections. Maybe the reviewer is asking about the incidence of cancer in general in these patients; several patients were treated for colon cancer, breast cancer, prostate cancer, skin cancer but this did not lead to their death. We did not collect the exact number of cancer cases in this study; we focused on mortality.

6. Reviewer’s comment: In page 10, Tables 1 and 2, the abbreviations should be indicated in the bottom of the tables.

Authors’ reply: Thank you for your comment. We amended the footnote accordingly.

7. Reviewer’s comment: In page 13, Table 3, the HR is calculated by the 1 unit increase or 1 SD increase of variables?

Authors’ reply: If we are assessing a continuous variable, HR indicates the change in the hazard of death if this variable changes by one unit; if the variable is categorical, HR reflects the hazard ratio of all-cause mortality by the presence of this variable.

8. Reviewer’s comment: In pages 14-16, Tables 4, 5, and 6, the authors can explain why age was not a risk for death in HD patients with diabetes?

Authors’ reply: Thank you for your comment. We were also surprised with this finding. We can only speculate that patients with diabetes have already a lot of cardiovascular complications and age wouldn’t have an impact.

9. Reviewer’s comment: In page 15 line 7, is the risk of a catheter for mortality shown in the tables? How many patients received the catheter HD?

Authors’ reply: No this was a sub-analysis of patients who died of infection-related mortality. Tables summarized the all-cause mortality. The percentage of patients dialyzed with a catheter was 11.2 % as depicted in Table 1.

10. Reviewer’s comment: In page 17 line 8, the elevation of ferritin ≥ 800 ng/mL is related to severe inflammation or presence of hemophagocytosis such as in malignant lymphoma and adult-onset Still disease?

Authors’ reply: No. Very high ferritin levels are common in hemodialysis patients and can be secondary to inflammation, past transfusions (we excluded patients with very frequent transfusions), iron overload.

11. Reviewer’s comment: In page 17 lines 20-23, are the differences in ferritin levels due to differences of dosage of iron supplementation?

Authors’ reply: it is mainly due to iron supplementation and past transfusions and probably to some inflammation. Hemodialysis patients have higher targets of ferritin than the general population.

**Reviewer #2:**

The authors try to reveal the predictive effects of Hb on mortality. The viewpoints of this study are new and attractive to clinicians. However, the analyses and data are preliminary. The authors need to clarify the following points.

1. Reviewer’s comment: It is unclear why the authors select 11g/dl as a cutoff level in this study. Can similar data be shown if the authors select 10 g/dl or 12 g/dl as a cutoff level? This is the very critical point of this study.

Authors’ reply: Thank you for your comment. We added to the statistical analysis the following: “Hemoglobin was assessed as a continuous variable in all analyses and as a categorical variable in the survival analysis by dividing patients into two groups based on the level of Hb < 11 or **≥** 11 g/dL. The threshold of 11 g/dL was selected taking into account that the target of hemoglobin in the latest guidelines varied between 10 and 12 g/dL”.

We would like to point out as well that our sample included a low percentage of patients with Hb<10 and Hb>12 which makes the comparison of these groups more difficult; it reflects as well the adherence of nephrologists to KDIGO guidelines.

2. Reviewer’s comment: Around 30% of the cause of death is infection. Inflammation should strongly affect iron metabolism and anemia. This is also a critical point of this study. The authors stated that they have no data about C-reactive protein as a limitation. If so, the result and conclusion are very weak and unacceptable. The authors need to adjust or evaluate the inflammatory factor in this study. A count of white blood cells might be one candidate for that.

Authors’ reply: Thank you for your comment. Our study took the average of Hb, ferritin and TSAT of all months and years of follow-up. This average cannot explain the death by infection that occurred at the end of follow-up and patients cannot be chronically infected for years and not die. Inflammation is not always secondary to infection. In addition, if high ferritin is due to inflammation rather than iron overload, the TSAT would be <20% (functional iron deficiency). The average TSAT in our study was 26%. However, we agree with the reviewer that adjustment to CRP might have been useful and this was mentioned in the limitations. We did not collect the white blood cell count in this study and it wouldn’t be useful to assess inflammation.

3. Reviewer’s comment: Control levels of diabetes are a key point for infection and/or cardiovascular events. Therefore, control levels of diabetes, such as glycoalbumin, are required. Moreover, SGLT2 inhibitors participate a good role in cardiovascular events. The data on the usage of SGLT2 inhibitors also require.

Authors’ reply: Patients included in this study are hemodialysis patients and so far, SGLT2 inhibitors are contra-indicated in this group of patients. We did not collect HbA1c for this study.

4. Reviewer’s comment: Microvascular or macrovascular damage is also a key factor in cardiovascular events. These data, such as retinopathy or ABI/PWV(ankle-brachial index/pulse wave velocity), are required.

Authors’ reply: Thank you for your comment. These variables were not collected.

**Reviewer #3:**

The authors compared the effect of renal anemia on mortality between diabetic and non-diabetic HD patients. Although the results were partially meaningful, there were several problems especially in study design and statistical analysis.

1. Reviewer’s comment: First, they presupposed the linear association between lower Hb levels and higher mortality. However, higher Hb levels could be also associated with worth outcome among CKD or dialyzed patients. I recommend using analysis with more detailed categories or restricted cubic spline models.

Authors’ reply: Thank you for your comment. We agree with the reviewer that higher hemoglobin levels are associated with worse outcomes. Several studies have already demonstrated the deleterious impact of Hb above 13 g/dL and all guidelines agreed that no patient should be targeted above 13. Accordingly, the vast majority of our patients were treated targeting a Hb between 10 and 12 g/dL. This has been shown in the amended Table 2, where the percentage of patients with Hb above 12 was 7.9%. We focused mainly in this study on comparing patients with and without diabetes and there is no difference in the Hb categories between these two groups of patients.

2. Reviewer’s comment: Second, they compared survival time between diabetic and non-diabetic patients. It is not suitable, because survival time is depend on observational duration. They recruited patients initiating HD between 2012 and 2020 and the follow-up period ended in 2021. The descriptions of comparisons of survival time in the Result section and Table 1 is not necessary.

Authors’ reply: Survival analysis assesses the time until the occurrence of an event. Patients who survived above the duration of the study were censored (right-censored). It is true that the survival time of these patients is incomplete but survival analysis models use methods of estimation that incorporate information from censored and uncensored observation.

We removed the description of survival times.

3. Reviewer’s comment: Third, thenumber of confounding factors were too much compared with low number ofoutcomes in multivariate analysis.

Authors’ reply: We agree with the reviewer, this is why we tried to group confounding variables into different models.

4. Reviewer’s comment: Minor comments
   Introduction
   P.4 L.13: The authors cited the findingsfrom TREAT study. It is not suitable, because this study included only non-dialyzedCKD patients with diabetes. Instead, I recommend citing the post hoc analysisof the Normal Hematocrit Cardiac Trial [Kilpatrick RD et al. Clin J Am SocNephrol 3: 1077–1083, 2008], because this study assessed the ESA responsivenessamong HD patients.

Authors’ reply: We know that TREAT did not include dialysis patients however TREAT influenced the guidelines worldwide to recommend lower levels of hemoglobin. The publication of Kilpatrick et al assessed the erythropoietin responsiveness and this was not our research topic.

5. Reviewer’s comment:
   P.5, L.13: The authors described that oneof the background of this study was that data on anemia and mortality in HDpatients are very scarce in countries from the Eastern Mediterranean region. Are the clinical pictures of dialysis or anemia different in this region from Europe, the US and Japan?

Authors’ reply: We answered to this question in the discussion where we compared our findings to the findings in these three regions.

6. Reviewer’s comment:
   Materials and Methods
   P.7, L.18: The authors described that “Continuousvariables are reported as mean ± standard deviation (SD) if normallydistributed and as medians with interquartile range (IQR) for variables notnormally distributed” in the Statistical analysis. It is well-known that serumferritin or PTH are not-normally distributed. Please describe as median and IQRand use non-parametrical test. There is no need to describe the type of test, tindependent test, Chi Square test, Mann Whitney U test or Fischer's exact test,in the Tables.

Authors’ reply: Thank you for your comment. We assessed the normal distribution of variables based on the histogram and the skewness test and we used the statistical tests accordingly.

7. Reviewer’s comment:
   P.8, L1: Only Log rank test is necessaryfor the assessment of Kaplan Meier survival analysis.
   Results
   I recommend combining Table 1 and Table 2together.

Authors’ reply: Thank you for your suggestion. This would lead to a long table and we would like to emphasize the laboratory results.

8. Reviewer’s comment:
   Discussion
   Discussion regarding the associationbetween higher mortality and age, serum albumin and PTH is too long.

Authors’ reply: Thank you for your comment. We agree that the paragraph is too long. We divided it into two paragraphs and removed some sentences and restructured the content.
